# Reports on the Hospitals of the United Kingdom

**Published:** 1910-03-19

**Authors:** Henry Burdett


### REPORTS
### ON THE
### Hospitals of the United Kingdom.

By SIR HENRY BURDETT, K.C.B., K.C.V.O.

### XXXII.—WARNEFORD, LEAMINGTON, AND SOUTH WARWICKSHIRE GENERAL HOSPITAL.

This institution was established in 1832, and since we inspected it in the nineties it has been enlarged and practically rebuilt. It has accommodation for 132 beds, but the pressure upon them has never been excessive, and for economical reasons the average number occupied rarely exceeds seventy. At this hospital, where nurses are trained and a private nursing staff is maintained, the matron, Miss Rapson, who was unfortunately absent, has for many years held that a hospital of this size should be self-contained, *i.e.* that the sisters should be selected from the nurses trained at the institution. It is interesting, therefore, in inspecting it to notice if Miss Rapson is justified in the course she has consistently maintained by the general efficiency of the whole hospital. We found it in excellent condition throughout. Indeed, many of the ward kitchens were in a high state of efficiency.

In the older portions of the building, which have recently been put in order, the space is too cramped, and most of the fittings are old. Good organisation has minimised the objections and difficulties caused by these defects, and it is but fair to say that Miss Rapson has justified her contention by the present high state of cleanliness and smartness which the hospital presents as a whole.

Our attention was particularly attracted by the fact that most of the porcelain baths were cracked and that some of them leaked. In our view, the hospital committee should make a strong representation to the architect and the firm from whom the baths were obtained, with the object of having these defective baths replaced at little or no cost to the hospital.

The establishment includes a good mortuary, a fever and isolation cottage hospital, and a nurses' home, where each nurse is provided with a separate bedroom. In the visitors' book we pointed out that it was desirable that a patients' lift should be provided without delay. We are glad to be able to report that the money for such a lift was speedily provided, a proof of the energy of the committee which it is a pleasure to record.

We had the privilege of meeting Miss Cumming, the assistant matron, who was trained here and who evidently takes the deepest interest in everything calculated to promote the efficiency of the hospital.

### XXXIII.—STRATFORD-ON-AVON HOSPITAL.

This hospital was rebuilt on a similar plan to that of the Johnson Hospital, Spalding, Lincolnshire, in 1884. We had not seen it for years, and looked forward to our inspection because the plan was a good deal criticised at the time it was built. On the whole, it has borne the test well, for, although faulty and expensive to work, it is comfortable and airy for the patients. It would not be fair to be too severe on the management as a change in the office of matron had just taken place, and the new matron had not yet taken over the duties. We thought it a good opportunity to make a careful inspection, and our conclusions are set forth in the following report which was entered in the visitors' report book:—

"I visited this hospital, after twelve years, with interest. It seems to me to present evidence of the need of internal organisation and general smartness. The nurses' room at the end of the long wards should be furnished more comfortably, as the present chairs are unsuitable for a duty room.

"The mortuary does not exhibit that respect for the dead or justice to the living which the hospital has the privilege to teach.

"It is certainly a good opportunity for a matron of ability to bring this hospital to a higher state of efficiency in several directions."

In addition to the supply of suitable furniture that the small day-rooms of the nurses require, a place should be found downstairs for the splint cupboard which should be removed from the nurses' recreation-room upstairs, where it is practically inaccessible. The box-room, too, should be cleared out and confined entirely to boxes, as at present it is little better than a lumber-room, and is not available for its original purpose. The charge of the instruments and the theatre, including the instruments used for post-mortems, should be given to the sister in charge of the surgical wards. It is quite wrong to place such matters in the hands of a night nurse who ought to be in bed when many operations are usually performed. Flock pillows, and, indeed, all bedding except feather pillows and hair mattresses, should be strictly excluded from use in this and all hospitals.

It is a great pity that arrangements have not been made to have a private nursing staff, for in these days every hospital should increase its voluntary income by giving to its subscribers the guarantee of an efficient trained nurse when needed and so attach the old subscribers to the institution, whilst many new subscribers should in this way be recruited and the annual income from voluntary sources be materially increased. This is an important business point which every hospital of importance is making in self-defence for economical and financial reasons.

**The Huddersfield Infirmary** will be reported on next week.

